# Association of Anti-GT1a Antibodies with an Outbreak of Guillain-Barré Syndrome and Analysis of Ganglioside Mimicry in an Associated *Campylobacter jejuni* Strain

**DOI:** 10.1371/journal.pone.0131730

**Published:** 2015-07-21

**Authors:** Maojun Zhang, Michel Gilbert, Nobuhiro Yuki, Fangfang Cao, Jianjun Li, Hongying Liu, Qun Li, Fanliang Meng, Jianzhong Zhang

**Affiliations:** 1 State Key Laboratory for Infectious Disease Prevention and Control, National Institute for Communicable Disease Control and Prevention, Chinese Center for Disease Control and Prevention, Beijing, China, 102206; 2 Collaborative Innovation Center for Diagnosis and Treatment of Infectious Diseases, Hangzhou, China, 310003; 3 Human Health Therapeutics, National Research Council Canada, Ottawa, K1A 0R6, Canada; 4 National Neuroscience Institute, Singapore, Singapore, 308433; 5 Office for Disease Control and Emergency Response, Chinese Center for Disease Control and Prevention, Beijing, 102206, China; The Ohio State University, UNITED STATES

## Abstract

An outbreak of Guillain-Barré syndrome (GBS), subsequent to *Campylobacter jejuni* enteritis, occurred in China in 2007. Serum anti-ganglioside antibodies were measured in GBS patients and controls. Genome sequencing was used to determine the phylogenetic relationship among three *C*. *jejuni* strains from a patient with GBS (ICDCCJ07001), a patient with gastroenteritis (ICDCCJ07002) and a healthy carrier (ICDCCJ07004), which were all associated with the outbreak. The ganglioside-like structures of the lipo-oligosaccharides of these strains were determined by mass spectrometry. Seventeen (53%) of the GBS patients had anti-GT1a IgG antibodies. GT1a mimicry was found in the lipo-oligosaccharides of strain ICDCCJ07002 and ICDCCJ07004; but a combination of GM3/GD3 mimics was observed in ICDCCJ07001, although this patient had anti-GT1a IgG antibodies. A single-base deletion in a glycosyltransferase gene caused the absence of GT1a mimicry in ICDCCJ07001. The phylogenetic tree showed that ICDCCJ07002 and ICDCCJ07004 were genetically closer to each other than to ICDCCJ07001. *C*. *jejuni*, bearing a GT1a-like lipo-oligosaccharide, might have caused the GBS outbreak and the loss of GT1a mimicry may have helped ICDCCJ07001 to survive in the host.

## Introduction

Guillain-Barré syndrome (GBS) is currently the most frequent cause of acute flaccid paralysis worldwide, since the near elimination of poliomyelitis [1]. Two-thirds of cases are preceded by symptoms of upper respiratory tract infection or gastrointestinal infections [2]. The most frequently identified infectious agent is *Campylobacter jejuni*, with 31% of infections being attributed to it in one systematic review [3]. Lipo-oligosaccharide (LOS) is a major component of the outer membrane of this bacterium; there is molecular mimicry between human gangliosides and *C*. *jejuni* LOS [4]. Infection by *C*. *jejuni*, bearing ganglioside-like LOS, induces the development of anti-ganglioside IgG antibodies in certain patients with *C*. *jejuni* infections [5]. The anti-ganglioside antibodies bind to gangliosides such as GM1 and GD1a, which are strongly expressed at the nodes of Ranvier, and activate the complement system, leading to the formation of membrane-attached complexes at the nodal axolemma of peripheral motor fibres. This results in the disappearance of voltage-gated sodium channels at the nodes and the disruption of axo-glial junctions, followed by a failure of motor nerve conduction and muscle weakness [6,7]. The synthesis of ganglioside-like LOS in *C*. *jejuni* usually requires three essential genes: either *cst-II* (encoding either a mono-functional α2,3-sialyltransferase or a bi-functional α2,3/8-sialyltransferase) or *cst-III* (encoding a mono-functional α2,3-sialyltransferase), *cgtA* (encoding a β-1,4-*N*-acetylgalactosaminyltransferase) and *cgtB* (encoding a β-1,3-galactosyltransferase). A strong association was found between the simultaneous presence of these three genes and GBS-associated *C*. *jejuni* strains [8,9]. These genes are present in 6 of the 22 LOS biosynthesis classes (A, B, C, M, R and V) that have been characterized in *C*. *jejuni* strains [10,11]. In addition, the class A locus was detected in 53% to 68% of the GBS associated isolates [9,12]. However, strains having the same class of LOS locus can express different ganglioside mimics because of DNA sequence polymorphisms in the *cst-II*, *cgtA* and *cgtB* genes, in addition to other genes encoding glycosyltransferases, involved in the biosynthesis of the LOS outer core [12,13,14]. For example, amino acid sequence variation in Cst-II affected its acceptor specificity. Strains with *cst-II* (Thr51) produce ganglioside mimics containing only α-2,3-linked sialic acid (NeuAc) residues such as GM1- and GD1a-like LOS. In contrast, strains with *cst-II* (Asn51) produce ganglioside mimics containing both α-2,3- and α-2,8-linked NeuAc residues such as GT1a-, GD3-like and GD1c-like LOS [13,15,16].

GBS is generally observed as a sporadic disease, but there have been few outbreaks reported [17,18]. In 2011, a *Campylobacter* infection outbreak, which included 26 identified cases of GBS, occurred at the USA-Mexico border in Yuma County (Arizona, USA) and San Luis Rio Colorado (Sonora, Mexico) [19]. The largest ever reported GBS outbreak occurred in Jilin (northern China) in June and July 2007, and *C*. *jejuni* infection was identified as the triggering factor [20]. One *C*. *jejuni* strain was isolated from a patient with GBS following a diarrhea episode (ICDCCJ07001), one strain was isolated from a patient with diarrhea only (ICDCCJ07002), and one strain was isolated from a healthy carrier (ICDCCJ07004), with the latter two strains being isolated from neighbors of the patients with GBS [20]. The genome sequences for ICDCCJ07001, 07002 and 07004 were recently determined [21,22].

A previous study using a commercial kit reported that most patients with GBS in the Jilin outbreak had anti-GM1 IgG antibodies [18]; however, repeated serological examinations using the same reagents indicated that these results were non-conclusive. In addition, the serum from a patient with GBS did not have significant immune reaction with the LOS from its associated strain (ICDCCJ07001) but had a strong reaction with the LOS from strain ICDCCJ07002, which was isolated from a patient with diarrhea only (data shown in the Results section). In order to further explore the pathogenesis of the GBS outbreak in northern China, we carried out a more thorough study that included the examination of anti-ganglioside antibodies in the sera of related populations using better standardized reagents and protocol [16,23,24], the structural determination of ganglioside-like LOS of *C*. *jejuni* strains from different hosts and the analysis of the genetic relatedness between these strains.

## Materials and Methods

### Ethics statement

The verbal informed consent for the blood sample collection from the patients in this study during the outbreak was obtained and the data was analyzed anonymously. Verbal informed consent for sample collection is permitted by the Chinese Center for Disease Control and Prevention (China CDC) for emergency outbreak investigation and the consent was approved by the ethics committee of the China CDC and the academic committee in the National Institute for Communicable Disease Control and Prevention. All the related documents were recorded at the China CDC. Ethics approval for this study was also obtained from the ethics committee of the China CDC and the academic committee of the National Institute for Communicable Disease Control and Prevention.

### Bacterial strains and serum samples

Strain ICDCCJ07001 was the unique isolate (single colony) isolated from the stool sample of one GBS patient who had preceding diarrhea. Strain ICDCCJ07002 was one colony picked among multiple colonies obtained following the culture from the stool sample of the neighbor of the GBS patient who had diarrhea at that time and strain ICDCCJ07004 was also one colony picked among multiple colonies obtained following the culture from the stool sample of a neighbor who had no diarrhea within the last 30 days [20].

During the outbreak of GBS in China in 2007, a total of 189 serum samples were obtained from patients with GBS subsequent to *C*. *jejuni* enteritis (n = 32), family members who had had *C*. *jejuni* enteritis (n = 12), neighbors who had had *C*. *jejuni* enteritis (n = 99) and healthy subjects (n = 46). The 32 serum samples from the GBS patients were taken from the patients when they first registered in the hospital between June 23 and July 9 (the first-phase collection) [20]. The family members’ serum samples were collected from the sister or brother of 12 individual GBS patients who had diarrhea during the same period as the GBS patients. The 99 serum samples from the neighbors were collected from the neighbors of the 32 GBS patients who shared the same water supply system and had diarrhea simultaneously with the GBS patients. Both the sample sets from the family members and from the neighbors were collected between June 23 and July 14. The healthy control samples were selected randomly from the stock of the local health inspection center over a period from January to June 2007. The subjects of the healthy control cohort did not have any underlying illness and were attending the health inspection center for regularly scheduled health checkups.

### Anti-LOS serology

Reactivity between the LOS extracted from *C*. *jejuni* ICDCCJ07001 and ICDCCJ07002 and the IgG antibodies in the serum from GBS patients (32 samples) and the controls (30 samples, 15 from the diarrheal patients and 15 from the healthy subjects) was measured with a modified enzyme-linked immunosorbent (ELISA) protocol. Briefly, LOS fractions from the *C*. *jejuni* strains were extracted by the hot aqueous-phenol method [25]. Both coomassie brilliant blue staining and silver staining were performed following separation of the samples by polyacrylamide gel electrophoresis in the presence of sodium dodecyl sulfate. The bands were visualized only with the silver stain which confirmed that there was no protein contamination. Each well was coated with 0.25 μg of purified LOS and serum samples were diluted 1:500 in PBS with 0.5% casein. Horseradish peroxidase-conjugated goat anti-human IgG antibodies (Signa-Aldrich, #A0170) was added as the secondary antibody. The captured antibodies were detected using tetramethyl benzidine as substrate by measuring the optical density (OD) at 450 nm.

### Anti-ganglioside serology

IgG and IgM antibodies against GM1, GM1b, GD1a, GD1b, GalNAc-GD1a, GT1a and GQ1b were measured in the 189 serum samples by an ELISA assay. The reagents were from by Dr. Yuki’s laboratory and the protocols were described previously [24]. Differences in the antibody frequencies among the groups were evaluated using the Fisher exact test with a statistical software package (IBM SPSS Statistics 19.0, Chicago, IL, USA). The level of significance was set at *p* < 0.05.

### Comparative genomics and phylogenetic analysis

The genome sequences of *C*. *jejuni* ICDCCJ07001, 07002 and 07004 were published previously [21,22] and were downloaded from GenBank (GenBank accession numbers NC_014802 for ICDCCJ07001, APNP00000000 for ICDCCJ07002 and APNQ00000000 for ICDCCJ07004). Comparative genomics and the core-genome single-nucleotide polymorphisms (SNPs) screen were performed by in silico analysis based on the genome sequence data. Briefly, we remapped all the assembled contigs to the completed reference genome sequence (strain ICDCCJ07001) to delineate shared regions with identity ≥ 90% and e-value <1e-5 according to BLASTn. Then we compared the raw reads of each strain to the core-genome by using SOAPaligner. SNPs were identified by aligning contigs of each strain to the genome of ICDCCJ07001 using MUMmer (version 3.22). The MUMmer results for each strain were filtered to remove SNPs that might be unreliable according to the following criteria: 1) quality scores <20 (average base calling error rate greater than 0.01); 2) covered by < 10 paired-end reads; 3) in repetitive regions; 4) not identified by BLAT searches of the contigs of each strain to core-genome sequences [26]. Qualified SNPs from ICDCCJ07001, 07002 and 07004 were verified using the BLAT (v 34) software to verify the matches from the alignments. DNA insertions and deletions shorter than 10 bp were extracted with axtBest and verified with BWA (version 0.5.8). In order to explain the evolutionary history of *C*. *jejuni* ICDCCJ07001, 07002 and 07004, a phylogenetic tree was constructed based on the core-genome concatenated SNPs using the software PHYML with the HKY model and 500 bootstrap replications [27,28]. To provide an outgroup for rooting the phylogenetic tree, two additional isolates were included in the phylogenetic analysis: *C*. *jejuni* 260.94, a strain isolated from a patient with GBS in Cape Town, South Africa [29] (GenBank accession number: NZ_AANK00000000), and *C*. *jejuni* HN-CJD07035, a strain isolated from a patient with diarrhea in the province of Henan, China (GenBank accession number: ARYE00000000). These two strains were selected because they are both Penner serotype HS:41, i.e. similar to ICDCCJ07001 and 07002 (07004 was untypable).

DNA sequence differences observed between the *cgtA*, *cgtB* and *cst-II* genes were confirmed by PCR amplication and DNA sequencing using primers cgtA-F (5’-AATTAATTTTTAGGTATAATC-3’), cgtA-R (5’-AAGAACAAAATTAATGGTTAC-3’), cgtB-F (5’-GAATTTAAAAAATTCTATTTAC-3’), cgtB-R (5’-CCATCAAGATTTATTTTTAACG-3’, cst-II-F (5’-GAAATTTTAAACATATTTATTC-3’) and cst-II-R (5’- CATTATGATTAATGCCTATTTC-3’).

### Mass spectrometry analysis

The LOS was extracted by the hot aqueous-phenol method as described previously [25]. Intact LOS samples were analyzed by capillary electrophoresis-electrospray ionization mass spectrometry, as described previously [30,31]. The LOS outer core structures were proposed based on the observed mass species and the presence of the glycosyltransferase variants in the LOS biosynthesis locus of the strains [14].

## Results

### Anti-LOS antibodies

**Fig 1:**
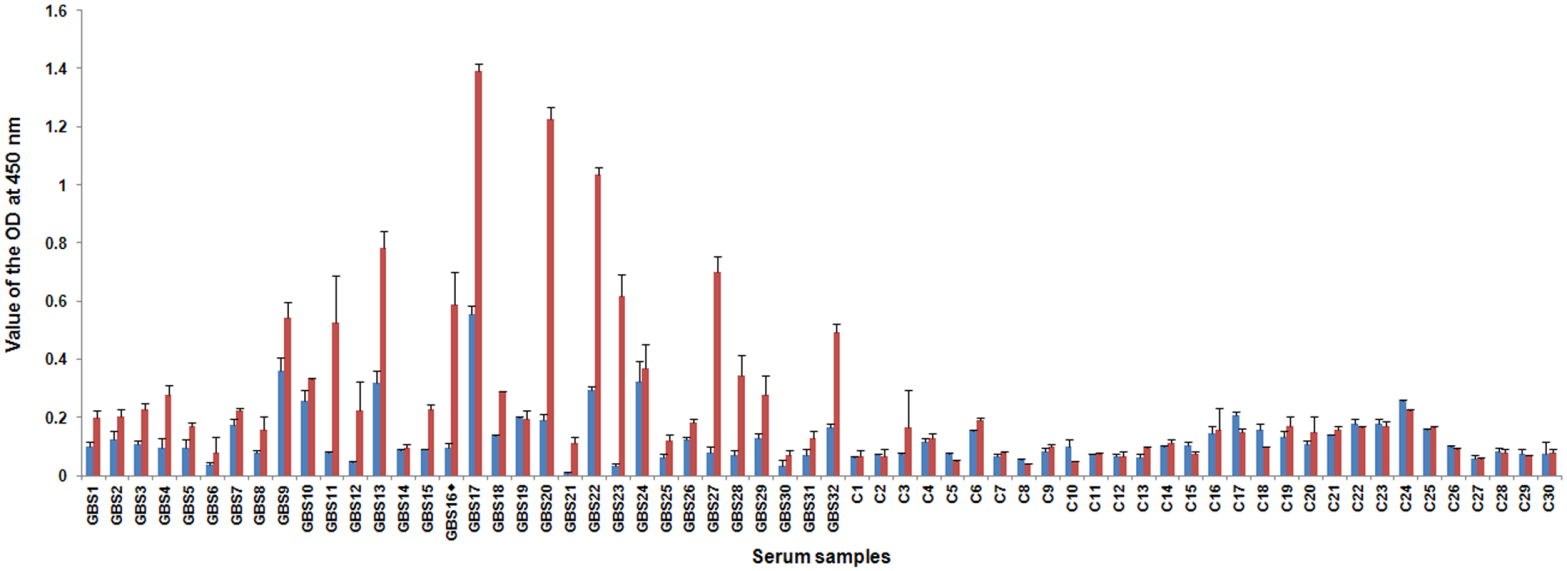
Anti-LOS IgG antibodies. Reactivity between the LOS extracted from *C*. *jejuni* ICDCCJ07001 and ICDCCJ07002 and the IgG antibodies in the sera from 32 GBS patients and 30 controls. The IgG antibodies in the sera from the GBS patients had stronger reactions with the LOS from *C*. *jejuni* ICDCCJ07002 than with the LOS from strain ICDCCJ07001 (the x-axis indicates the ID of the serum samples and the y-axis indicates the value of the OD at 450 nm_;_ red bar for anti-07002 LOS reaction and blue bar for anti-07001 LOS reaction. The first 32 serum samples were from GBS patients and the last 30 samples were used as controls, 15 from the diarrheal patients and 15 from the healthy subjects). The serum sample GBS16 (labeled with a black diamond) corresponds to the patient from whom *C*. *jejuni* ICDCCJ07001 was isolated.

The IgGs in the sera from the GBS patients had stronger reactions with the LOS from *C*. *jejuni* ICDCCJ07002 than with the LOS from strain ICDCCJ07001 (Fig 1, the x-axis indicates the ID of the serum samples and the y-axis indicates the value of the OD at 450 nm_;_ red bar for anti-07002 LOS reaction and blue bar for anti-07001 LOS reaction). IgG antibodies in the serum sample from GBS16 (labeled with a black diamond), corresponding to the patient from whom *C*. *jejuni* ICDCCJ07001 was isolated, had a strong reaction with the LOS from strain ICDCCJ07002 (OD value at 450 nm of 0.589) but not with the LOS from strain ICDCCJ07001 (OD value at 450 nm of 0.096).

### Anti-ganglioside antibodies

In total, 20 (63%) of the 32 patients with GBS had IgG antibodies against any of the gangliosides tested (Table 1). IgG antibodies against GM1, GD1b, GT1a and GQ1b were detected in four (13%), one (3%), 17 (53%) and two (6%) of the patients, respectively. The frequency of the anti-GT1a antibodies was significantly higher in the GBS group than in the other groups (*p* < 0.001). Among the 17 patients with GBS who had anti-GT1a antibodies, 14 had no antibodies against the other gangliosides, whereas two had anti-GQ1b antibodies, and one had anti-GM1 antibodies. The serum from the patient with GBS, from whom *C*. *jejuni* ICDCCJ07001 was isolated, had the highest titre of anti-GT1a antibodies (OD = 3.05) among the entire set of tested samples. Only three samples had positive anti-GT1a IgM antibodies among the 189 tested samples. Two of them were from GBS patients and were positive for both anti-GT1a IgG and IgM antibodies. One was from the neighbor group and had only anti-GT1a IgM antibodies. Positive frequencies of IgM antibodies did not show significant difference among those four groups.

**Table 1:** Anti-ganglioside IgG antibodies.

					Two-tailed *p*-value		
	GBS	FM	N	HC	GBS vs FM	GBS vs N	GBS vs HC
Number	32	12	99	46			
GM1	4 (13%)	1 (8%)	1 (1%)	0	NS	0.016	0.025
GM1b	0	0	1 (1%)	1 (2%)	NS	NS	NS
GD1a	0	0	0	0	NS	NS	NS
GalNAc-GD1a	0	0	0	0	NS	NS	NS
GD1b	1 (3%)	0	0	0	NS	NS	NS
GT1a	17 (53%)	0	2 (2%)	0	0.004	<0.001	<0.001
GQ1b	2 (6%)	0	0	0	NS	NS	NS
Any of the gangliosides above	20 (63%)	1 (8%)	4 (4%)	1 (2%)	<0.001	<0.001	<0.001

Abbreviations: GBS = Guillain-Barré syndrome subsequent to *C*. *jejuni* enteritis; FM = family members who had had *C*. *jejuni* enteritis; N = neighbors who had had *C*. *jejuni* enteritis; HC = healthy controls; NS = not significant (*p* > 0.05).

### Comparative genomics and DNA sequencing of *cgtA*, *cgtB* and *cst-II* in *C*. *jejuni* ICDCCJ07001, 07002 and 07004

Only small DNA insertions, deletions and SNPs were found among ICDCCJ 07001, 07002 and 07004 when their whole genome sequences were compared. The similarity of these three isolates at the genome level was more than 99%. The genome alignment results and the locations of the SNPs detected among these three isolates are shown in S1 Fig. Three genes (*cgtA*, *cgtB* and *cst-II*) of the LOS biosynthesis locus were amplified and re-sequenced to confirm their expression status. The *cgtB* and *cst-II* genes were intact (no premature translational stop codon) and had identical sequences in ICDCCJ07001, 07002 and 07004. The three *cgtA* versions were nearly identical, with the only difference being a single bp deletion in ICDCCJ07001 (A136), which caused a frame-shift mutation and premature translational stop codon after 57 amino acids. Therefore, ICDCCJ07001 carried a version of *cgtA* that encoded an inactive β-1,4-*N*-acetylgalactosaminyltransferase.

### Phylogenetic relations based on the core genome SNPs

In total, 134 qualified core-genome SNPs were discovered among ICDCCJ07001, 07002 and 07004 (S1 Table). Seventy-six of the 134 SNPs were located within genes; the other 58 were in the intergenic regions. Of the SNPs occurring within genes, 65 were non-synonymous changes (nsSNPs) and 11 were synonymous changes (sSNPs; Table 2).

**Table 2:** Number of core-genome SNPs unique to each *Campylobacter jejuni* strain.^a^

Strain	sSNP^b^	nsSNP^c^	Intergenic	Total
ICDCCJ07001	11	61	57	129
ICDCCJ07002	0	2	1	3
ICDCCJ07004	0	2	0	2

Abbreviations: SNP = single-nucleotide polymorphism

^a^The SNPs are listed in S1 Table

^b^Synonymous SNP

^c^Non-synonymous SNP

**Fig 2:**
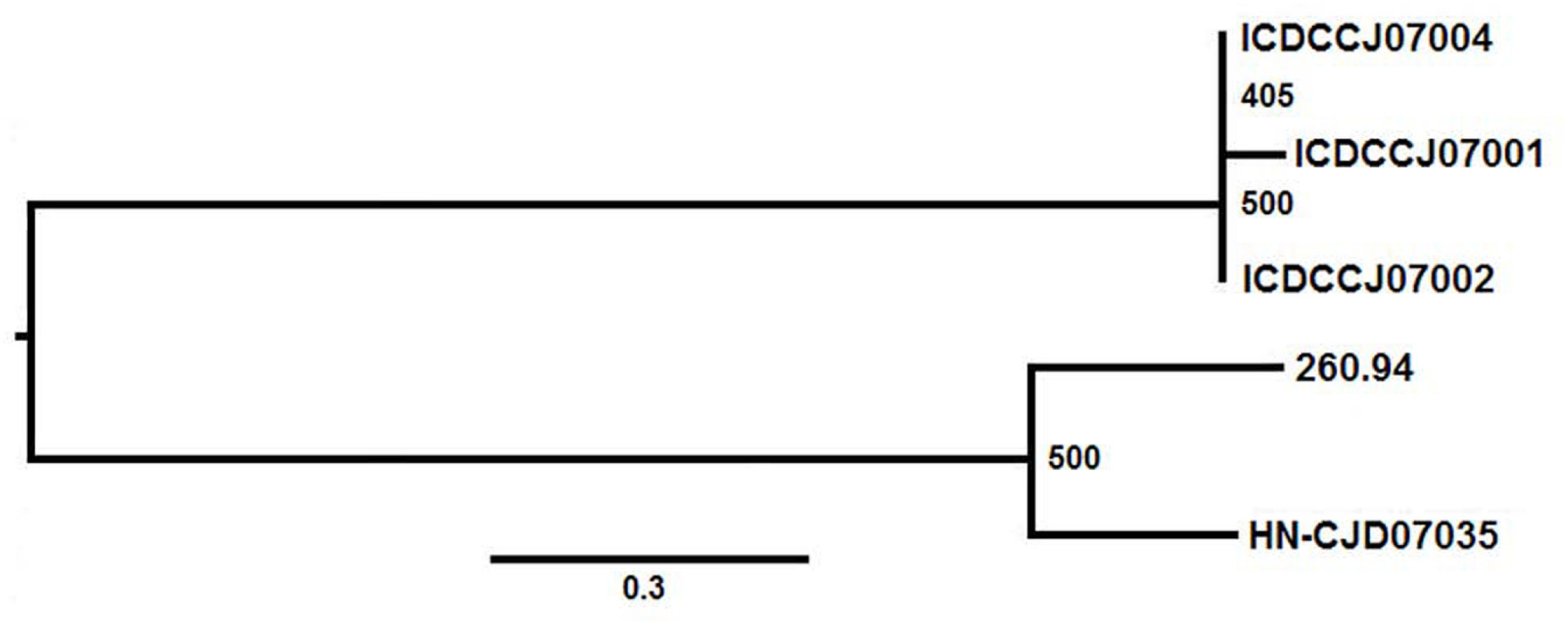
Phylogenetic relatedness of 5 *C*. *jejuni* isolates. The maximum likelihood phylogenetic tree showing the relatedness of 5 *C*. *jejuni* strains was based on the core-genome SNPs and created using the software PHYML in the treeBest software package with the HKY model and 500 bootstrap replications. Numbers at the branches are bootstrap values and the branch lengths correlate with the numbers of SNPs between the strains.

A phylogenetic tree, based on the 2348 core-genome SNPs from the three isolates and *C*. *jejuni* isolates 260.94 and HN-CJD07035, was created using the software PHYML in the treeBest software package with the HKY model and 500 bootstrap replications (Fig 2). The maximum bootstrap value of the phylogenetic tree was 500, which indicated the reliability of the relationships. By including the two outgroup strains, the phylogenetic analysis indicated that the three isolates from the outbreak study belonged to the same cluster. The tree also indicated that ICDCCJ07002 and 07004 were genetically closer to each other than to ICDCCJ07001. The analysis of the SNPs suggested that ICDCCJ07002 and 07004 were closer than ICDCCJ07001 to the two “external” HS:41 strains (260.94 and HN-CJD07035).

### Ganglioside-like structure of the LOS

**Fig 3:**
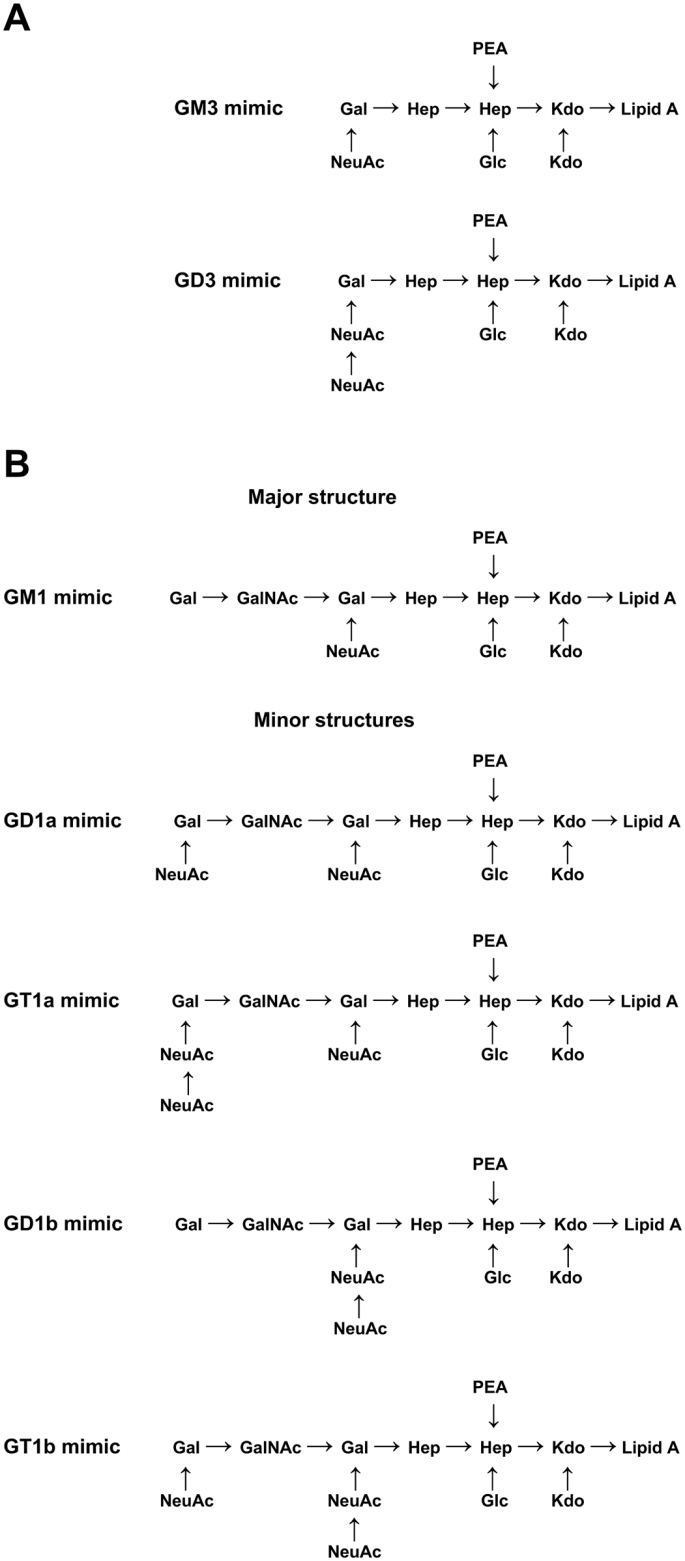
Proposed LOS outer core structures based on capillary electrophoresis-electrospray ionization mass spectrometry. **(A)** LOS outer core structures determined for *C*. *jejuni* ICDCCJ07001. **(B)** LOS outer core structures determined for both *C*. *jejuni* ICDCCJ07002 and 07004. The major structure corresponds to mass species having the highest intensities in the mass spectra of the LOS samples.

Mass spectrometry analysis of an intact LOS sample from ICDCCJ07001 suggested an outer core composition including two hexoses and one NeuAc residue (Hex_2_NeuAc_1_), along with some mass species variants having two NeuAc residues (Hex_2_NeuAc_2_; Fig 3A and S2 Table). The presence of di-NeuAc was confirmed by the observation of a fragment ion at m/z 581.5 when tandem mass spectrometry was carried out on the triply charged ion at m/z 1285.4 (data not shown). The glycosyltransferase variants present in the LOS locus of ICDCCJ07001 were predicted to have similar specificities to those of *C*. *jejuni* OH4382 [32]. The key glycosyltransferases are the bi-functional Cst-II (Asn51) sialyltransferase and a truncated version of the β-1,4-*N*-acetylgalactosaminyltransferase (CgtA). The bi-functional Cst-II (Asn51) is responsible for adding an α-2,3-linked NeuAc to the terminal galactose (Gal) residue, and then an α-2,8-linked NeuAc to the first NeuAc residue. The *cgtA* version present in ICDCCJ07001 has a 7 A tract at position 129–135 (rather than the 8 A tract present in active *cgtA* versions) which causes a frame-shift mutation and premature translational stop after 57 amino acids. The presence of a truncated and inactive CgtA prevents further elongation beyond the Gal residue that is sialylated. The analysis of the glycosyltransferase variants, combined with the mass spectrometry data, suggests that ICDCCJ07001 expresses a combination of GM3 and GD3 mimics in the outer core region of its LOS (Fig 3A).

Mass spectrometry analysis of intact LOS samples of ICDCCJ07002 and 07004 showed that both strains have identical outer cores (Fig 3B and S2 Table). The mass spectrum of ICDCCJ07004 gave peaks with stronger relative intensities and was studied in further detail. The most abundant ions in the ICDCCJ07004 spectrum corresponded to mass species with a proposed outer core composition of three hexoses, one *N*-acetylhexosamine and one NeuAc residue (Hex_3_HexNAc_1_NeuAc_1_; S2 Table). Mass species variants having one or two additional NeuAc residues (Hex_3_HexNAc_1_NeuAc_2_ and Hex_3_HexNAc_1_NeuAc_3_) were also observed. The presence of di-NeuAc in the ion at m/z 1407.1 (Hex_3_HexNAc_1_NeuAc_2_) and in the ion at m/z 1504.2 (Hex_3_HexNAc_1_NeuAc_3_) was confirmed by the observation of a fragment ion at m/z 581.5 using tandem mass spectrometry (data not shown). However, it was not possible to determine if the ion at m/z 1407 contained only mass species with di-NeuAc as a chain, or if there was a mix with mass species that also contained two mono- NeuAc residues on separate Gal residues. The glycosyltransferase variants present in the LOS loci of ICDCCJ07002 and 07004 were predicted to have similar specificities as the ones in *C*. *jejuni* OH4384 [13,32]. The glycosyltransferase variants in ICDCCJ07002 and 07004 are consistent with the major observed outer core composition of Hex_3_HexNAc_1_NeuAc_1_ corresponding to a GM1 mimic (Fig 3B). Cst-II (Asn51) can add an α-2,3-linked NeuAc to the Gal residue that is attached to the heptose residue. CgtA and CgtB will extend the outer core by adding a β-1,4-linked *N*-acetylgalactosamine residue and a β-1,3-linked Gal residue, respectively, resulting in a GM1 mimic. Further extension by transfer of one or two additional NeuAc residues would be performed by Cst-II (Asn51) which is able to add both an α-2,3-linked NeuAc to the terminal Gal residue and an α-2,8-linked NeuAc to the α-2,3-linked NeuAc residue. The ions with the additional NeuAc residues were much less abundant than ions corresponding to the GM1 mimic. Consequently, we propose that the di-sialylated mimics (GD1a and GD1b) and tri-sialylated mimics (GT1a and GT1b) are minor structures present in the LOS of ICDCCJ07002 and 07004 (Fig 3B).

## Discussion

In the present study, we found that half of the 32 patients with GBS had anti-GT1a IgG antibodies without GQ1b reactivity. Monospecific anti-GT1a antibodies are associated with pharyngeal-cervical-brachial weakness, a localized subtype of acute motor axonal neuropathy, an axonal variant of GBS [23,33]. Previous studies showed that 15 (47%) of the 32 patients with GBS had acute motor axonal neuropathy, according to the electro-diagnostic criteria [20,34,35]. It could be because pharyngeal-cervical-brachial weakness remains unfamiliar to many neurologists, and also being an axonal variant of GBS, which could present similar symptoms as the motor axonal neuropathy during the later stage [33], that this specific clinical diagnosis was not made at that time. According to the anti-gangliosides antibodies results, there is a possibility that the outbreak in Jilin might be the first pharyngeal-cervical-brachial weakness outbreak. This is clinically important because the clinical manifestation of pharyngeal-cervical-brachial weakness is also similar to that of botulism, which is a foodborne illness with occasional outbreaks around the world [36,37]. Examination for the specific anti-ganglioside antibodies present in patient sera is crucial for establishing the correct diagnosis based on the identified symptoms.

*C*. *jejuni* strain ICDCCJ07001 was isolated from a GBS patient who had breathing and swallowing difficulties at the onset of the illness, which was also classified as an axonal neuropathy [20]. The serum from this patient had the highest titre of anti-GT1a IgG antibodies, but did not have significant immune reaction with the LOS from its associated strain (ICDCCJ07001). Unexpectedly, it had a strong reaction with the LOS from strain ICDCCJ07002, which was isolated from a patient with diarrhea only. The difference in serum reactivity suggested that *C*. *jejuni* ICDCCJ07001 and 07002 strains expressed different ganglioside-like structures and that the ICDCCJ07002 strain had a GT1a-like structure. The LOS structural differences between ICDCCJ07001 (GM3/GD3 mimics) and 07002 (GM1/GD1a/GD1b/GT1a/GT1b mimics) provide an explanation for the difference in serum reactivity. GM3/GD3 mimics are truncated versions of the GM1/GD1a/GD1b/GT1a/GT1b mimics, resulting from an inactive CgtA β-1,4-*N*-acetylgalactosaminyltransferase. The inactivation of *cgtA* in ICDCCJ07001 resulted in a truncated LOS mimicking GM3 and GD3, which was not reactive with the corresponding patient serum. In order to exclude that the inactivation of *cgtA* occurred during in vitro passaging, we compared its sequence in different ICDCCJ07001 sub-cultures and did not find any differences.

There is a possibility that the inactivation of *cgtA* could have been triggered by pressure from the immune response against the GT1a mimic or by the selection of a sublineage with an inactive *cgtA* already present in the *C*. *jejuni* population. This would be a form of antigenic drift, which in this case has resulted in a change of the ganglioside mimics being presented on the surface of the cells, which could have extended the survival of the strain in this patient. Analyzing a larger number of strains would have allowed a more thorough investigation but it is extremely difficult to collect a comprehensive set of GBS associated and control isolates because of the delay between the occurrence of the enteritis outbreak and the onset of neurological symptoms among a sub-group of patients who frequently have cleared the infection by then. In this case, the median interval from diarrhea onset to neurological symptom onset was 10 days (range: 5 to 20 days) [20,38].

There are two other reports of closely related pairs of strains that have LOS loci that differed only by the deletion of a single A base in their respective *cgtA* genes [10]. Two of these strains (OH4382 and OH4384) were clearly epidemiologically related as they came from Japanese siblings [39]. The inactivation of this gene seems to have a significant clinical role, as it has been observed in at least three unrelated pairs of cases.

Strain ICDCCJ07001 was isolated from a patient with GBS who had been hospitalized 7 days after the neurological disorder and diarrhea had occurred. The persisting status of this GBS strain is in contrast with the outcome observed with an outbreak of *C*. *jejuni* enteritis in a Dutch family where *C*. *jejuni* could be isolated from two family members with enteritis only, but could not be isolated from the family member who developed GBS following diarrhea [40]. The patient with GBS was the only family member who had a strong immune response against gangliosides (GM1 and asialo-GM1 in this case) and against the LOS from the *C*. *jejuni* isolates from the other two family members.

Another case of antigenic drift of a carbohydrate antigen (lipopolysaccharide) was reported for *Shigella flexneri* [41]. Serotype conversion due to a single missense mutation was observed using *S*. *flexneri* isolates which were recovered from an infected patient over a period of 39 days. Serotype conversion would have enhanced the survival of the strain in this patient since immunity to *S*. *flexneri* is serotype specific.

The large number of unique and non-synonymous SNPs in ICDCCJ07001, compared with ICDCCJ07002 and 07004, suggests that this strain was under higher pressure from the immune response in the patient from whom *C*. *jejuni* ICDCCJ07001 was isolated. The 61 non-synonymous SNPs unique to ICDCCJ07001 are located within 43 coding sequences (S1 Table) which encode 9 hypothetical proteins and 34 proteins that have homology with members from various functional categories including motility accessory factors, surface polysaccharide biosynthesis proteins, outer membrane proteins and putative transporters (see S1 Table for other putative annotations). One SNP resulted in a nonsense mutation in a hypothetical protein while the majority of the other non-synonymous SNPs resulted in non-conservative amino acid substitutions. It is difficult to predict the effect of these amino acid substitutions on the functions of the affected proteins. However, motility and cell surface structures are known to have an impact on the adaptability to the environment and virulence of bacterial strains, so it is possible that some of the non-synonymous SNPs had an impact on the survival of ICDCCJ07001.

In summary, the present study provides a comprehensive analysis of the genetic relatedness and pathogenesis of *C*. *jejuni* isolates obtained during the course of the largest GBS outbreak ever reported.

## Supporting Information

S1 Fig Comparison of the genomes from *Campylobacter jejuni* ICDCCJ07001, 07002 and 07004. The genome of ICDCCJ07001 is used as the reference and shown as a black ring. The purple and blue rings represent the genomes of ICDCCJ07002 and ICDCCJ07004, respectively. The locations of the SNPs are marked with vertical bars outside of the circles. The detected SNPs are listed in S1 Table.
(TIF)

S1 Table List of the 134 core-genome SNPs detected in *Campylobacter jejuni* ICDCCJ07001, 07002 and 07004. (DOC)

S2 Table Mass spectrometry data and proposed compositions for intact LOS of *Campylobacter jejuni* strains. (DOC)
