# Supplementary figures and images for: Association of Anti-GT1a Antibodies with an Outbreak of Guillain-Barré Syndrome and Analysis of Ganglioside Mimicry in an Associated Campylobacter jejuni Strain
Source: PLoS One. 2015 Jul 21;10(7):e0131730. doi: 10.1371/journal.pone.0131730 (PMC4510130; doi:10.1371/journal.pone.0131730)

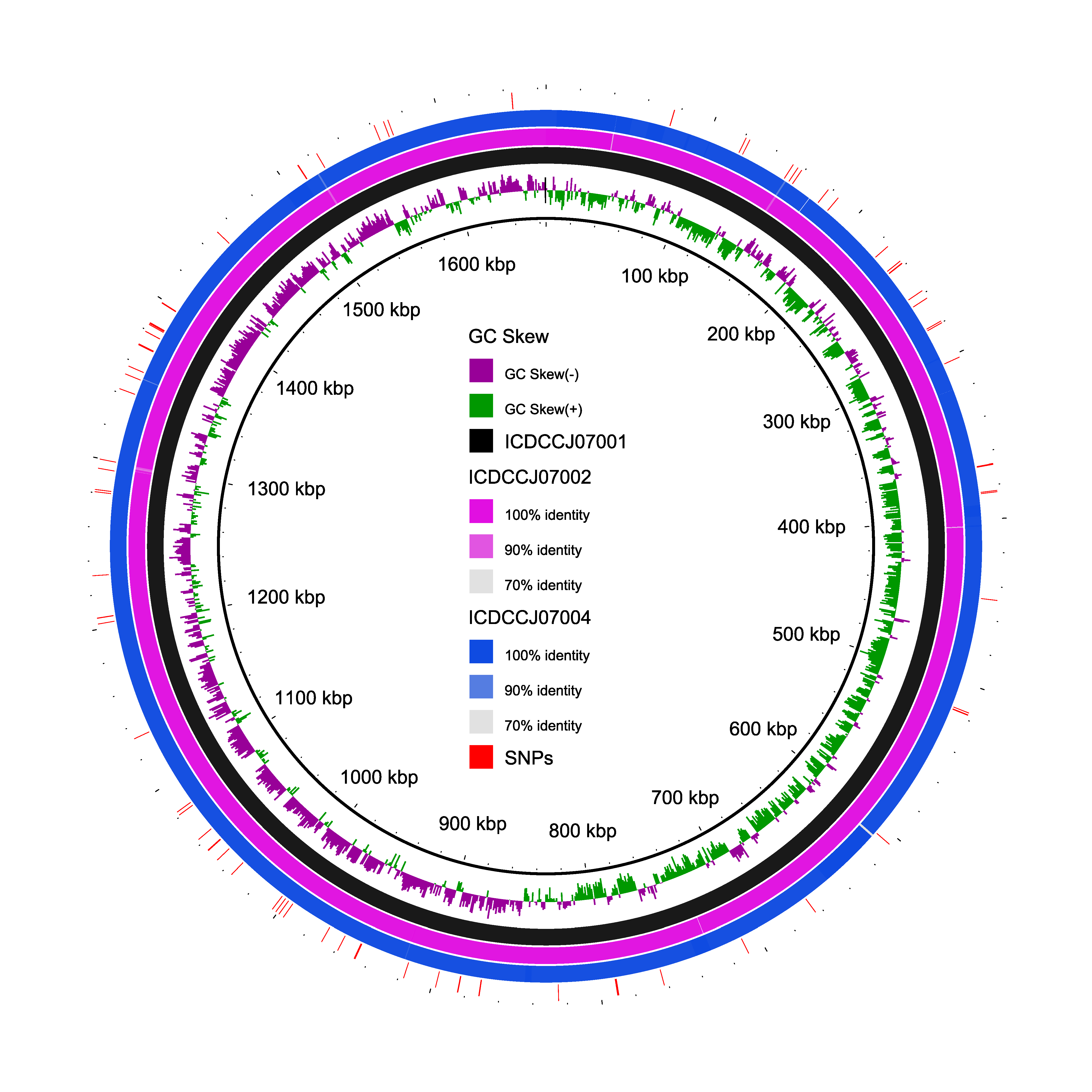

Supplement: S1 Fig — The genome of ICDCCJ07001 is used as the reference and shown as a black ring. The purple and blue rings represent the genomes of ICDCCJ07002 and ICDCCJ07004, respectively. The locations of the SNPs are marked with vertical bars outside of the circles. The detected SNPs are listed in S1 Table. (TIF) [file pone.0131730.s001.tif]
