# Supplementary material for: Association of Anti-GT1a Antibodies with an Outbreak of Guillain-Barré Syndrome and Analysis of Ganglioside Mimicry in an Associated Campylobacter jejuni Strain
Source: PLoS One. 2015 Jul 21;10(7):e0131730. doi: 10.1371/journal.pone.0131730 (PMC4510130; doi:10.1371/journal.pone.0131730)
Supplement: S1 Table — (DOC) [file pone.0131730.s002.doc]

**S1 Table. List of the 134 core-genome SNPs detected in *Campylobacter jejuni* ICDCCJ07001, 07002 and 07004**

| **SNP position on the genome of ICDCCJ07001** | **SNPs in different strains** | | | **Gene tag in ICDCCJ07001** | **MutationType** | **ICDCCJ07001 AA** | **Mutation AA** | **Protein Product** |
| --- | --- | --- | --- | --- | --- | --- | --- | --- |
|  | **07001** | **07002** | **07004** |  |  |  |  |  |
| 75959 | G | T | T | ------- | intergenic | - | - | - |
| 75960 | T | G | G | ------- | intergenic | - | - | - |
| 76045 | T | A | A | ------- | intergenic | - | - | - |
| 120844 | C | A | A | ICDCCJ07001_100 | nonsyn | F | L | ATP synthase subunit B |
| 123249 | G | T | T | ------- | intergenic | - | - | - |
| 152414 | T | C | C | ------- | intergenic | - | - | - |
| 155732 | C | A | A | ICDCCJ07001_141 | nonsyn | T | N | hypothetical protein |
| 158314 | A | T | T | ------- | intergenic | - | - | - |
| 158316 | C | T | T | ------- | intergenic | - | - | - |
| 158317 | A | T | T | ------- | intergenic | - | - | - |
| 158319 | A | T | T | ------- | intergenic | - | - | - |
| 158322 | C | G | G | ------- | intergenic | - | - | - |
| 158327 | C | T | T | ------- | intergenic | - | - | - |
| 158328 | C | T | T | ------- | intergenic | - | - | - |
| 177560 | A | A | C | ICDCCJ07001_170 | nonsyn | V | G | iron-uptake ABC transport system periplasmic iron-binding protein |
| 185032 | T | C | C | ICDCCJ07001_177 | nonsyn | V | A | transporter |
| 225548 | C | A | A | ICDCCJ07001_222 | nonsyn | T | N | mechanosensitive ion channel family protein |
| 236699 | C | G | G | ------- | intergenic | - | - | - |
| 236701 | T | A | A | ------- | intergenic | - | - | - |
| 236702 | T | A | A | ------- | intergenic | - | - | - |
| 236705 | T | A | A | ------- | intergenic | - | - | - |
| 236710 | T | A | A | ------- | intergenic | - | - | - |
| 236711 | C | A | A | ------- | intergenic | - | - | - |
| 238259 | T | C | C | ICDCCJ07001_233 | nonsyn | I | T | hypothetical protein |
| 238410 | C | T | T | ICDCCJ07001_233 | syn | - | - | hypothetical protein |
| 238492 | G | T | T | ICDCCJ07001_234 | nonsyn | K | N | hypothetical protein |
| 238493 | G | T | T | ICDCCJ07001_234 | nonsense | E | * | hypothetical protein |
| 258216 | T | A | A | ------- | intergenic | - | - | - |
| 263357 | C | A | A | ------- | intergenic | - | - | - |
| 279010 | C | A | A | ICDCCJ07001_274 | nonsyn | M | I | molybdenum ABC transporter, permease |
| 280110 | T | T | C | ------- | intergenic | - | - |  |
| 302631 | A | G | G | ICDCCJ07001_300 | nonsyn | R | G | hypothetical protein |
| 367776 | T | A | A | ------- | intergenic | - | - | - |
| 367781 | T | C | C | ------- | intergenic | - | - | - |
| 367824 | G | T | T | ------- | intergenic | - | - | - |
| 367917 | G | T | T | ------- | intergenic | - | - | - |
| 368197 | T | A | A | ------- | intergenic | - | - | - |
| 368198 | T | A | A | ------- | intergenic | - | - | - |
| 368199 | T | A | A | ------- | intergenic | - | - | - |
| 383545 | A | T | T | ICDCCJ07001_376 | nonsyn | M | L | GTP-binding protein |
| 383547 | G | T | T | ICDCCJ07001_376 | nonsyn | M | I | GTP-binding protein |
| 384318 | C | T | T | ICDCCJ07001_377 | nonsyn | L | F | hypothetical protein |
| 447908 | G | A | G | ICDCCJ07001_442 | nonsyn | G | D | DNA-directed RNA polymerase, beta' subunit |
| 515761 | C | A | A | ICDCCJ07001_503 | nonsyn | S | I | gamma-glutamyl phosphate reductase |
| 516988 | G | T | T | ICDCCJ07001_505 | nonsyn | W | L | pyridine nucleotide-disulfide oxidoreductase family protein |
| 517163 | G | C | C | ICDCCJ07001_505 | syn | - | - | pyridine nucleotide-disulfide oxidoreductase family protein |
| 517164 | T | G | G | ICDCCJ07001_505 | nonsyn | Y | D | pyridine nucleotide-disulfide oxidoreductase family protein |
| 517213 | T | C | C | ICDCCJ07001_505 | nonsyn | F | S | pyridine nucleotide-disulfide oxidoreductase family protein |
| 605609 | C | A | A | ICDCCJ07001_600 | nonsyn | R | S | di-/tripeptide transporter |
| 664190 | A | C | C | ICDCCJ07001_654 | nonsyn | C | G | hypothetical protein |
| 709922 | T | C | C | ICDCCJ07001_702 | nonsyn | V | A | periplasmic solute-binding protein |
| 709923 | C | T | T | ICDCCJ07001_702 | syn | - | - | periplasmic solute-binding protein |
| 762594 | A | T | T | ICDCCJ07001_748 | nonsyn | L | H | valyl-tRNA synthetase |
| 789746 | A | G | G | ICDCCJ07001_774 | nonsyn | I | M | cysteinyl-tRNA synthetase |
| 789949 | G | T | T | ICDCCJ07001_775 | nonsyn | K | N | cysteinyl-tRNA synthetase |
| 790418 | A | G | G | ICDCCJ07001_775 | nonsyn | N | D | cysteinyl-tRNA synthetase |
| 825023 | T | A | A | ------- | intergenic | - | - | - |
| 825035 | A | T | T | ------- | intergenic | - | - | - |
| 871556 | T | G | G | ICDCCJ07001_852 | nonsyn | H | P | phenylalanyl-tRNA synthetase, beta subunit |
| 871782 | C | A | A | ICDCCJ07001_852 | nonsyn | A | S | phenylalanyl-tRNA synthetase, beta subunit |
| 871783 | A | T | T | ICDCCJ07001_852 | nonsyn | N | K | phenylalanyl-tRNA synthetase, beta subunit |
| 884195 | A | T | T | ICDCCJ07001_866 | nonsyn | F | L | O-acetylserine lyase |
| 884227 | C | T | T | ------- | intergenic | - | - | - |
| 884233 | A | C | C | ------- | intergenic | - | - | - |
| 884237 | T | A | A | ------- | intergenic | - | - | - |
| 884256 | C | A | A | ------- | intergenic | - | - | - |
| 884268 | A | T | T | ------- | intergenic | - | - | - |
| 884295 | A | G | G | ------- | intergenic | - | - | - |
| 884346 | A | T | T | ------- | intergenic | - | - | - |
| 884437 | G | T | T | ------- | intergenic | - | - | - |
| 884445 | T | A | A | ------- | intergenic | - | - | - |
| 897527 | C | A | A | ------- | intergenic | - | - | - |
| 916917 | G | T | T | ICDCCJ07001_897 | syn | - | - | 2-acylglycerophosphoethanolamine acyltransferase / acyl-acyl carrier protein synthetase |
| 947366 | T | G | G | ICDCCJ07001_933 | nonsyn | S | A | periplasmic protein |
| 947367 | C | T | T | ICDCCJ07001_933 | nonsyn | S | L | periplasmic protein |
| 947892 | A | T | T | ICDCCJ07001_933 | nonsyn | E | V | periplasmic protein |
| 947893 | A | G | G | ICDCCJ07001_933 | syn | - | - | periplasmic protein |
| 947894 | T | C | C | ICDCCJ07001_933 | nonsyn | Y | H | periplasmic protein |
| 958582 | T | A | A | ------- | intergenic | - | - | - |
| 969222 | G | C | C | ICDCCJ07001_953 | nonsyn | H | D | porphobilinogen synthase |
| 995663 | A | T | T | ICDCCJ07001_982 | nonsyn | I | K | cytochrome c family protein |
| 998798 | C | T | T | ICDCCJ07001_985 | nonsyn | R | K | sigma-54 dependent DNA-binding response regulator |
| 998810 | C | T | T | ICDCCJ07001_985 | nonsyn | R | K | sigma-54 dependent DNA-binding response regulator |
| 1001085 | G | A | A | ICDCCJ07001_988 | syn | - | - | DNA gyrase, A subunit |
| 1001086 | A | G | G | ICDCCJ07001_988 | nonsyn | V | A | DNA gyrase, A subunit |
| 1003571 | G | T | T | ICDCCJ07001_990 | nonsyn | T | N | lipoprotein |
| 1034799 | C | T | T | ICDCCJ07001_1019 | nonsyn | R | K | sulfatase domain protein |
| 1046671 | C | A | A | ICDCCJ07001_1029 | nonsyn | P | T | membrane-associated zinc metalloprotease |
| 1054275 | C | A | A | ICDCCJ07001_1037 | nonsyn | F | L | hypothetical protein |
| 1054621 | G | A | A | ICDCCJ07001_1038 | nonsyn | R | K | periplasmic protein |
| 1062881 | G | A | A | ICDCCJ07001_1046 | nonsyn | S | L | folC bifunctional protein |
| 1081045 | C | A | A | ------- | intergenic | - | - | - |
| 1083150 | G | A | A | ------- | intergenic | - | - | - |
| 1083151 | C | A | A | ------- | intergenic | - | - | - |
| 1132706 | C | A | A | ------- | intergenic | - | - | - |
| 1203052 | C | G | G | ICDCCJ07001_1182 | syn | - | - | glycyl-tRNA synthetase, beta subunit |
| 1206432 | T | A | A | ICDCCJ07001_1184 | nonsyn | Y | N | hypothetical protein |
| 1206557 | T | A | A | ICDCCJ07001_1185 | syn | - | - | phosphatase |
| 1231404 | G | A | A | ------- | intergenic | - | - | - |
| 1280522 | T | C | C | ICDCCJ07001_1255 | nonsyn | V | A | CMP-N-acetylneuraminic acid synthetase |
| 1281834 | C | A | A | ------- | intergenic | - | - | - |
| 1281846 | C | T | T | ------- | intergenic | - | - | - |
| 1294358 | A | G | G | ICDCCJ07001_1270 | nonsyn | N | D | hypothetical protein |
| 1299621 | T | C | C | ICDCCJ07001_1273 | nonsyn | N | D | motility accessory factor |
| 1342288 | G | A | G | ICDCCJ07001_1310 | nonsyn | P | S | non-canonical purine NTP pyrophosphatase, RdgB/HAM1 family |
| 1351528 | G | T | T | ICDCCJ07001_1320 | nonsyn | P | Q | hypothetical protein |
| 1351529 | G | T | T | ICDCCJ07001_1320 | nonsyn | P | T | hypothetical protein |
| 1356473 | C | T | T | ICDCCJ07001_1325 | nonsyn | S | F | cryptic C4-dicarboxylate transporter DcuD |
| 1370224 | G | T | T | ------- | intergenic | - | - | - |
| 1370232 | G | A | A | ------- | intergenic | - | - | - |
| 1370754 | G | T | T | ICDCCJ07001_1338 | nonsyn | P | T | phosphomannomutase/phosphoglucomutase |
| 1379000 | T | A | A | ICDCCJ07001_1346 | syn | - | - | Na+/H+ antiporter |
| 1379047 | G | A | A | ICDCCJ07001_1346 | nonsyn | L | F | Na+/H+ antiporter |
| 1383547 | C | G | G | ICDCCJ07001_1351 | nonsyn | C | S | glycosyltransferase |
| 1384215 | A | C | C | ------- | intergenic | - | - | - |
| 1384248 | G | C | C | ------- | intergenic | - | - | - |
| 1384251 | A | T | T | ------- | intergenic | - | - | - |
| 1384301 | T | C | C | ------- | intergenic | - | - | - |
| 1384727 | T | C | C | ICDCCJ07001_1352 | nonsyn | K | E | sugar transferase |
| 1384754 | T | C | C | ICDCCJ07001_1352 | nonsyn | K | E | sugar transferase |
| 1398080 | T | G | G | ICDCCJ07001_1364 | nonsyn | K | Q | WbcB |
| 1398363 | C | A | A | ICDCCJ07001_1365 | nonsyn | R | S | dTDP-4-dehydrorhamnose 3,5-epimerase |
| 1398364 | C | T | T | ICDCCJ07001_1365 | nonsyn | R | K | dTDP-4-dehydrorhamnose 3,5-epimerase |
| 1398365 | T | A | A | ICDCCJ07001_1365 | nonsyn | R | W | dTDP-4-dehydrorhamnose 3,5-epimerase |
| 1450696 | T | C | C | ICDCCJ07001_1412 | syn | - | - | hypothetical protein |
| 1511722 | A | T | T | ICDCCJ07001_1469 | nonsyn | Y | N | helicase |
| 1511975 | G | T | T | ICDCCJ07001_1469 | nonsyn | D | E | helicase |
| 1524697 | C | T | T | ------- | intergenic | - | - | - |
| 1562056 | C | C | T | ICDCCJ07001_1520 | nonsyn | P | L | DNA-binding response regulator |
| 1567953 | G | C | C | ICDCCJ07001_1525 | nonsyn | M | I | TonB-dependent heme receptor |
| 1570631 | C | A | A | ICDCCJ07001_1528 | syn | - | - | hemin ABC transporter, periplasmic hemin-binding protein |
| 1644749 | C | A | A | ------- | intergenic | - | - | - |
| 1644870 | C | T | T | ------- | intergenic | - | - | - |
| 1644878 | C | T | T | ------- | intergenic | - | - | - |
