# Supplementary material for: Association of Anti-GT1a Antibodies with an Outbreak of Guillain-Barré Syndrome and Analysis of Ganglioside Mimicry in an Associated Campylobacter jejuni Strain
Source: PLoS One. 2015 Jul 21;10(7):e0131730. doi: 10.1371/journal.pone.0131730 (PMC4510130; doi:10.1371/journal.pone.0131730)
Supplement: S2 Table — (DOC) [file pone.0131730.s003.doc]

**S2 Table. Mass spectrometry data and proposed compositions for intact LOS of *C. jejuni* strains**

| **Strain** | **Observed ions**  **(*m/z*)** | | | **Molecular mass**  **(Da)** | | **Core composition** | **Phosphorylation of lipid A** | **Acylation in lipid A** |
| --- | --- | --- | --- | --- | --- | --- | --- | --- |
|  | **[M-5H]^5-^** | **[M-4H]^4-^** | **[M-3H]^3-^** | **Observed** | **Calculated ^1^** |  |  |  |
| ICDCCJ07001 |  | 883.9 | 1179.0 | 3539.8 | 3539.9 | NeuAc_1_•Hex_2_•Hep_2_•*P*Etn_1_•Kdo_2_•lipid A | *P*, *PP*Etn | 3 *N-*(3-OH-C_14:0_), 1 *O-*(3-OH-C_14:0_), 1*O-*(C_14:0_), 1*O-*(C_16:0_) |
|  |  | 891.0 | 1188.4 | 3568.1 | 3567.9 | NeuAc_1_•Hex_2_•Hep_2_•*P*Etn_1_•Kdo_2_•lipid A | *P*, *PP*Etn | 3 *N-*(3-OH-C_14:0_), 1 *O-*(3-OH-C_14:0_), 2*O-*(C_16:0_) |
|  |  | 921.7 | 1229.4 | 3691.0 | 3691.0 | NeuAc_1_•Hex_2_•Hep_2_•*P*Etn_1_•Kdo_2_•lipid A | *PP*Etn, *PP*Etn | 3 *N-*(3-OH-C_14:0_), 1 *O-*(3-OH-C_14:0_), 2*O-*(C_16:0_) |
|  |  | 956.7 | 1276.1 | 3831.1 | 3831.1 | NeuAc_2_•Hex_2_•Hep_2_•*P*Etn_1_•Kdo_2_•lipid A | *P*, *PP*Etn | 3 *N-*(3-OH-C_14:0_), 1 *O-*(3-OH-C_14:0_), 1*O-*(C_14:0_), 1*O-*(C_16:0_) |
|  | 770.9 | 963.8 | 1285.4 | 3859.3 | 3859.2 | NeuAc_2_•Hex_2_•Hep_2_•*P*Etn_1_•Kdo_2_•lipid A | *P*, *PP*Etn | 3 *N-*(3-OH-C_14:0_), 1 *O-*(3-OH-C_14:0_), 2*O-*(C_16:0_) |
|  |  | 987.2 | 1317.0 | 3953.4 | 3954.2 | NeuAc_2_•Hex_2_•Hep_2_•*P*Etn_1_•Kdo_2_•lipid A | *PP*Etn, *PP*Etn | 3 *N-*(3-OH-C_14:0_), 1 *O-*(3-OH-C_14:0_), 1*O-*(C_14:0_), 1*O-*(C_16:0_) |
|  | 795.5 | 994.6 | 1326.4 | 3982.4 | 3982.2 | NeuAc_2_•Hex_2_•Hep_2_•*P*Etn_1_•Kdo_2_•lipid A | *PP*Etn, *PP*Etn | 3 *N-*(3-OH-C_14:0_), 1 *O-*(3-OH-C_14:0_), 2*O-*(C_16:0_) |
| ICDCCJ07002 |  | 951.9 | 1268.8 | 3810.5 | 3810.2 | NeuAc_1_•HexNAc_1_•Hex_3_•Hep_2_•*P*Etn_1_•Kdo_2_•lipid A | *P*, *P* | 3 *N-*(3-OH-C_14:0_), 1 *O-*(3-OH-C_14:0_), 2*O-*(C_16:0_) |
|  |  | 982.3 | 1310.1 | 3933.3 | 3933.3 | NeuAc_1_•HexNAc_1_•Hex_3_•Hep_2_•*P*Etn_1_•Kdo_2_•lipid A | *P*, *PP*Etn | 3 *N-*(3-OH-C_14:0_), 1 *O-*(3-OH-C_14:0_), 2*O-*(C_16:0_) |
|  | 843.8 | 1055.2 | 1407.1 | 4224.4 | 4224.5 | NeuAc_2_•HexNAc_1_•Hex_3_•Hep_2_•*P*Etn_1_•Kdo_2_•lipid A | *P*, *PP*Etn | 3 *N-*(3-OH-C_14:0_), 1 *O-*(3-OH-C_14:0_), 2*O-*(C_16:0_) |
|  | 902.3 | 1128.1 | 1504.5 | 4516.5 | 4515.8 | NeuAc_3_•HexNAc_1_•Hex_3_•Hep_2_•*P*Etn_1_•Kdo_2_•lipid A | *P*, *PP*Etn | 3 *N-*(3-OH-C_14:0_), 1 *O-*(3-OH-C_14:0_), 2*O-*(C_16:0_) |
| ICDCCJ07004 |  | 975.4 | 1300.8 | 3905.5 | 3905.2 | NeuAc_1_•HexNAc_1_•Hex_3_•Hep_2_•*P*Etn_1_•Kdo_2_•lipid A | *P*, *PP*Etn | 3 *N-*(3-OH-C_14:0_), 1 *O-*(3-OH-C_14:0_), 1*O-*(C_14:0_), 1*O-*(C_16:0_) |
|  | 785.5 | 982.5 | 1310.1 | 3933.3 | 3933.3 | NeuAc_1_•HexNAc_1_•Hex_3_•Hep_2_•*P*Etn_1_•Kdo_2_•lipid A | *P*, *PP*Etn | 3 *N-*(3-OH-C_14:0_), 1 *O-*(3-OH-C_14:0_), 2*O-*(C_16:0_) |
|  |  | 1013.3 | 1351.0 | 4056.6 | 4056.3 | NeuAc_1_•HexNAc_1_•Hex_3_•Hep_2_•*P*Etn_1_•Kdo_2_•lipid A | *PP*Etn, *PP*Etn | 3 *N-*(3-OH-C_14:0_), 1 *O-*(3-OH-C_14:0_), 2*O-*(C_16:0_) |
|  | 843.9 | 1055.1 | 1407.1 | 4224.4 | 4224.5 | NeuAc_2_•HexNAc_1_•Hex_3_•Hep_2_•*P*Etn_1_•Kdo_2_•lipid A | *P*, *PP*Etn | 3 *N-*(3-OH-C_14:0_), 1 *O-*(3-OH-C_14:0_), 2*O-*(C_16:0_) |
|  | 896.6 | 1121.2 | 1494.8 | 4488.1 | 4487.7 | NeuAc_3_•HexNAc_1_•Hex_3_•Hep_2_•*P*Etn_1_•Kdo_2_•lipid A | *P*, *PP*Etn | 3 *N-*(3-OH-C_14:0_), 1 *O-*(3-OH-C_14:0_), 1*O-*(C_14:0_), 1*O-*(C_16:0_) |
|  | 902.2 | 1128.0 | 1504.2 | 4515.9 | 4515.8 | NeuAc_3_•HexNAc_1_•Hex_3_•Hep_2_•*P*Etn_1_•Kdo_2_•lipid A | *P*, *PP*Etn | 3 *N-*(3-OH-C_14:0_), 1 *O-*(3-OH-C_14:0_), 2*O-*(C_16:0_) |
|  | 926.8 | 1158.8 | 1545.3 | 4639.0 | 4638.8 | NeuAc_3_•HexNAc_1_•Hex_3_•Hep_2_•*P*Etn_1_•Kdo_2_•lipid A | *PP*Etn, *PP*Etn | 3 *N-*(3-OH-C_14:0_), 1 *O-*(3-OH-C_14:0_), 2*O-*(C_16:0_) |

^1^ Isotope-average mass units were used for calculation of molecular mass values based on proposed compositions as follows: Hex,162.14; HexNAc, 203.20; Hep, 192.17; Kdo, 220.18; P, 79.98; *P*Etn, 123.05; NeuAc, 291.26; C_14:0_, 210.36; C_16:0_, 238.41; HexN, 161.16; HexN_3_N, 160.18; 3‑OH‑C_14:0_, 226.36; H_2_O, 18.01.
